# Optimization of spray‐drying conditions for green acerola concentrate juice: effect of temperature and maltodextrin

**DOI:** 10.1002/jsfa.70354

**Published:** 2025-11-28

**Authors:** Fábia Sabino Costa, Jhenifer Cristina Carvalho Santos, Michelly Duarte Costa e Silva, Matheus de Souza Cruz, Amanda Aparecida de Lima Santos, Cassiano Rodrigues de Oliveira, Letícia Fernandes de Oliveira, Jefferson Luiz Gomes Corrêa

**Affiliations:** ^1^ Department of Food Science Federal University of Lavras Lavras Brazil; ^2^ Laboratory of Bioprocesses and Metabolic Biochemistry Federal University of São João del‐Rei Divinópolis Brazil; ^3^ Institute of Exact and Technological Sciences, Federal University of Viçosa Rio Paranaíba Brazil

**Keywords:** *Malpighia glabra*, *Malpighia emarginata*, response surface methodology, phenolic compounds, ascorbic acid, microencapsulation

## Abstract

**BACKGROUND:**

Acerola (*Malpighia glabra* and *M. emarginata*) is a tropical fruit predominantly cultivated in northeastern Brazil, notable for its abundance of health‐beneficial bioactive compounds, including ascorbic acid, phenolic compounds, flavonoids, and anthocyanins. However, due to its high perishability, preservation processes such as spray drying have become essential. Given its low acidity, high sugar content, and the presence of low‐molecular‐weight substances, acerola poses several challenges during drying, which require careful evaluation of process parameters. This study aimed to evaluate and optimize the spray‐drying process parameters, temperature and maltodextrin concentration, for the production of green acerola powder from concentrated juice.

**RESULTS:**

A central composite rotatable design was conducted, varying temperature (110–180 °C) and maltodextrin concentration (3–30%, w/w, based on total solids content of the concentrated juice). Water activity, yield, color parameters, ascorbic acid, antioxidant activity by DPPH (2,2‐diphenyl‐1‐picrylhydrazyl) and ABTS (2,2′‐azino‐bis(3‐ethylbenzothiazoline‐6‐sulfonic acid), total phenolic compounds, and hygroscopicity were analyzed as response variables. The best conditions for achieving higher yield, ascorbic acid retention, and antioxidant activity were a temperature of 145 °C and 16.5% maltodextrin. Increasing the maltodextrin concentration contributed to the reduction of water activity. Color was influenced by both the carrier concentration and temperature, and obtaining less hygroscopic powders was favored by increasing maltodextrin concentration and decreasing temperature. Overall, the results indicate that moderate drying temperatures combined with intermediate maltodextrin concentrations favor the preservation of ascorbic acid and antioxidant compounds, minimizing thermal degradation and oxidative losses during the drying process.

**CONCLUSION:**

Spray drying of green acerola juice concentrate under optimized conditions enables the production of powders with improved yield, higher retention of bioactive compounds, and reduced hygroscopicity, highlighting its potential as a viable method for preserving acerola's nutritional and functional qualities. © 2025 The Author(s). *Journal of the Science of Food and Agriculture* published by John Wiley & Sons Ltd on behalf of Society of Chemical Industry.

## INTRODUCTION

Acerola (*Malpighia glabra* and *M. emarginata*) is originally from the Caribbean and thrives in tropical and subtropical regions. It is recognized for its rich nutritional profile and the presence of several bioactive constituents, especially ascorbic acid (vitamin C), which occurs in remarkably high levels and holds significant commercial value, alongside phenolic compounds, flavonoids, and anthocyanins. The vitamin C content in some acerola varieties ranges from 15 to 45 g 100 g^−1^ dry basis, which is up to 100 times higher than that found in oranges.^1^, ^2^ Although acerola is recognized for its high nutritional value, its high perishability and seasonal nature constitute major challenges for its commercialization and consumption, resulting in substantial waste along the production chain.

Studies on the chemical variation during the ripening stages of acerola (immature and mature) revealed that ascorbic acid predominantly accumulates in immature fruits, which also show greater 2,2‐diphenyl‐1‐picrylhydrazyl (DPPH) and 2,2′‐azino‐bis(3‐ethylbenzothiazoline‐6‐sulfonic acid (ABTS) radical scavenging capacity compared to mature fruits. Therefore, further research is needed to explore the use of green acerola (immature fruit) due to its relevant technological properties, such as high vitamin C content and antioxidant activity.^3^, ^4^, ^5^, ^6^ Drying is an excellent preservation technique for extending shelf life, as it inhibits natural enzymatic activity and reduces microbial growth.^7^ A typical process used for many plant extracts is spray drying.^8^, ^9^ The spray dryer promotes the obtainment of powders from the dehydration of droplets by their quick contact with heated air in a process involving a high rate of heat and mass transfer.^10^, ^11^, ^12^

Despite the well‐established use of spray drying for ripe acerola,^13^, ^14^, ^15^ only two studies have investigated the drying of green acerola: Albuquerque Junior *et al*.,^16^ using convective drying, reported better preservation of bioactive compounds at 50 °C, while Teixeira *et al*.^6^ were the only ones to apply atomization, achieving approximately 88% retention of vitamin C and high antioxidant activity. This highlights a significant gap in the industrial application of green acerola.

Spray drying of foods rich in sugars and organic acids is challenging due to the tendency of these compounds to exhibit sticky behavior during drying. Two phenomena occur: particle–particle cohesion and particle–wall adhesion. This leads to product deposition on the equipment walls and issues with powder flow and recovery at the end of the process, resulting in losses.^17^, ^18^ This difficulty is related to the low glass transition temperature (*T*_g_) of these materials. When the process temperature exceeds *T*_g_, molecular mobility increases, leading to particle adhesion and stickiness. To address these challenges, it is essential to use an adjuvant in the drying process. The incorporation of high‐*T*_g_ carrier agents raises the matrix *T*_g_, reduces stickiness, and improves powder yield, being essential to stabilize such products during drying.^19^

Maltodextrin is widely applied in spray drying, recognized for its affordability and low moisture absorption capacity. Additionally, due to its well‐defined physical characteristics and easy dissolution in water, maltodextrin has become a commonly used additive in the food industry. It prevents lump formation and acts as a protective carrier, ensuring high retention (65–80%) of volatile compounds.^1^, ^12^ In dairy emulsions, O’Neill *et al*.^19^ observed that the partial replacement of lactose with maltodextrin significantly increased the powder *T*_g_ and reduced its stickiness, highlighting the crucial role of this carrier in improving product stability and handling properties.

In this context, the present study aimed to determine the optimal spray‐drying conditions – specifically, drying temperature and maltodextrin concentration – for processing concentrated green acerola juice, with the goal of producing a powder with improved drying performance and greater preservation of key bioactive components, such as ascorbic acid, total phenolics, and antioxidant activity, suitable for applications in the food, cosmetic, and pharmaceutical industries.

## MATERIALS AND METHODS

### Materials

**Figure 1:**
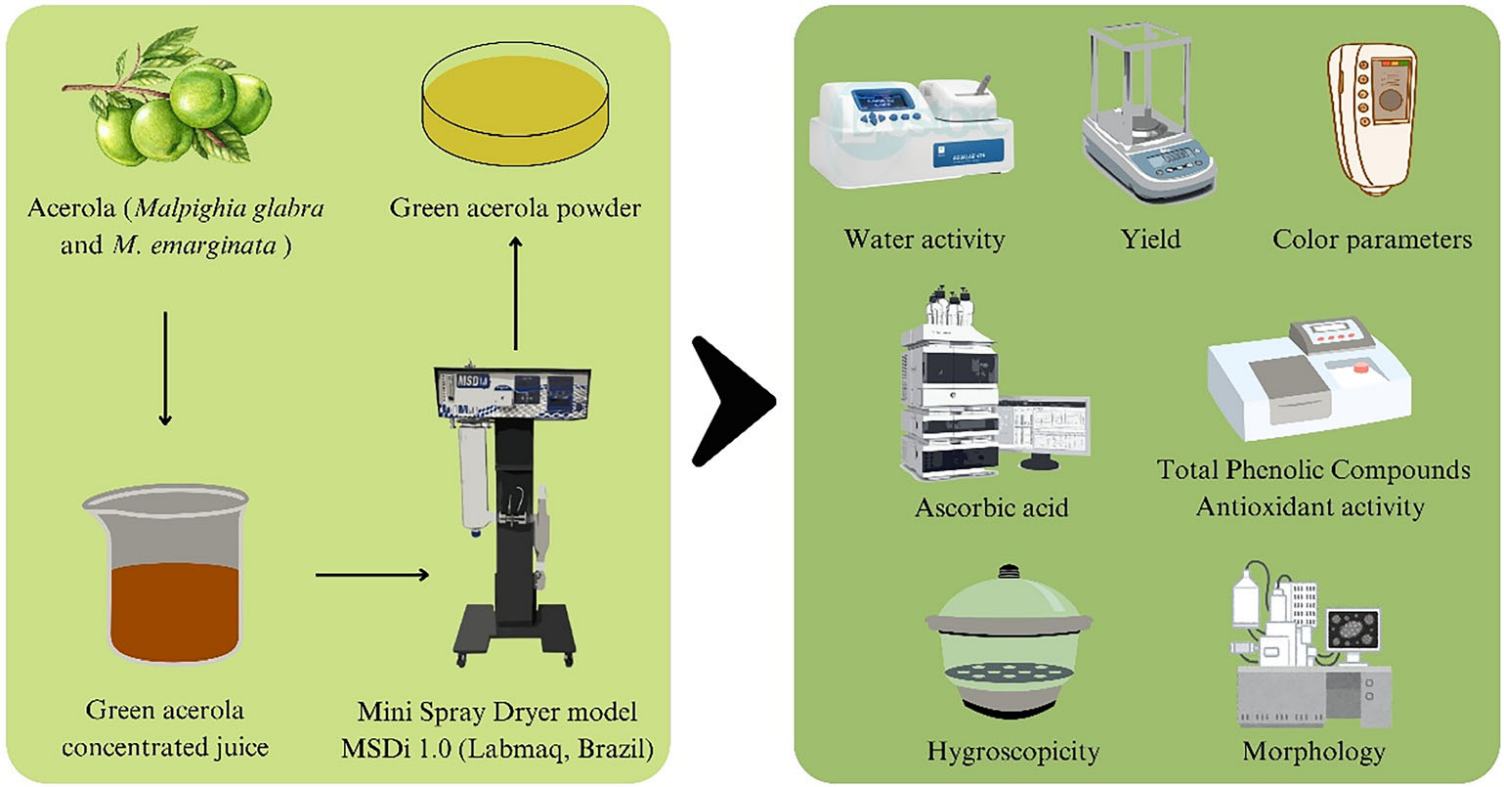
Graphical representation of the experimental planning.

Concentrated green acerola juice was obtained from the industrial processing of green acerola fruits (Amway Nutrilite Farm do Brasil Ltda, Ubajara, CE, Brazil). The juice had a total solids content of approximately 32% and a soluble solids content of 32 °Brix. This equivalence is due to the extract preparation process, which involved grinding, pressing, decantation, and ultrafiltration steps, resulting in a clarified extract predominantly composed of soluble solids. A single batch was used for all experiments to ensure uniformity and consistency of the samples. Samples were collected and stored at −15 °C, remaining refrigerated until the preparation of the solution according to the experimental design for its use in the subsequent stages (Fig. 1). The maltodextrin used was DE 10 Maltrin M100 from Grain Processing Group (Muscatine, IA, USA), and the calcium hydroxide was USP grade from Labsynth (Diadema, SP, Brazil).

### Spray drying

Spray drying was performed in a mini spray dryer (model MSDi 1.0, Labmaq, Ribeirão Preto, SP, Brazil), with the following parameters set: 1.0 mm diameter atomizing nozzle, compressed air pressure of 3.5 kgf cm^−2^, atomizing air flow rate of 40.5 L min^−1^, and feed flow rate of 0.45 L h^−1^.

The pH of the juice was adjusted to 7.0 using calcium hydroxide, as preliminary tests indicated higher drying yield under these conditions. The initial pH of the concentrated green acerola juice was approximately 3.85 ± 0.21, and its neutralization helped to reduce stickiness and wall deposition during spray drying, thereby improving process efficiency.^20^, ^21^ The concentrated green acerola juice (150 mL) was maintained under agitation for 30 min in a water bath at 45 °C. After preparation, the drying processes were carried out according to an experimental design based on a central composite rotatable design (CCRD). Each treatment was analyzed in triplicate, and the statistical analysis was based on the mean values, with the residual variance estimated from the replicated central points. The experimental design comprised 11 runs, structured factorially with five levels for the process variables: drying temperature and maltodextrin concentration (calculated based on the total solids content of the concentrated green acerola juice). The determination of these levels was grounded in literature data^22^, ^23^ and validated by preliminary tests, ensuring broad coverage of the study space. The reliability of the experimental error estimation was ensured by including three replicates at the central point (Table 1).

**Table 1:** Actual and coded independent variables of the CCRD of green acerola juice by spray drying

Temperature (°C)	110 (−1.41)	120 (−1)	145 (0)	170 (1)	180 (1.41)
Maltodextrin (%)	3.0 (−1.41)	7.0 (−1)	16.5 (0)	26.0 (1)	30.0 (1.41)

Coded values are in parentheses.

### Water activity (*a*_w_)

The water activity of was determined using an water activity meter (AquaLab 3TE model, Decagon Devices, São José dos Campos, SP, Brazil). The temperature was maintained at 24.5 ± 0.1 °C during the tests.

### Drying yield

The drying yield was calculated according to Cuevas‐Glory *et al*.^24^ It was evaluated by determining the product recovery, given by the percentage ratio between the total mass of the recovered product and the mass of the concentrated juice fed into the system on a dry basis.

### Color parameters

Color measurements were performed using a colorimeter (CM‐5 model, Konica Minolta, Tokyo, Japan) under the D65 standard illuminant, with a 10° standard observer and the specular component excluded (SCE), focusing on the parameters L* (lightness) and *h*° (hue angle) according to the CIE LCH color space system.

### Determination of ascorbic acid

The extraction of ascorbic acid was carried out based on the procedure proposed by Cruz *et al*.,^25^ with slight adaptations. Initially, 1 g of sample was mixed with 10 mL of a 4.5% metaphosphoric acid solution prepared in ultrapure water and left to stand for 1 h in an amber container to prevent oxidation. The mixture was then subjected to ultrasonic treatment for 30 min using an ultrasonic bath (USC 2850 A, Unique, Indaiatuba, SP, Brazil). After extraction, the sample was centrifuged at 7000 rpm for 10 min, and the supernatant was transferred to amber microtubes (1.5 mL). Quantification of ascorbic acid was performed using a high‐performance liquid chromatography system (LC‐20AT, Shimadzu, Kyoto, Japan) equipped with an ultraviolet (UV) detector (SPD‐20A, Shimadzu), following the method of Vinci *et al*.,^26^ with modifications. Chromatographic separation was achieved on a C18 column (5 μm, 250 × 4.6 mm; Phenomenex, Torrance, CA, USA), maintained at 30 °C. The mobile phase consisted of 0.15% (v/v) aqueous acetic acid, delivered at a constant flow rate of 1.0 mL min^−1^. The detection wavelength was set at 254 nm. Identification of the ascorbic acid peak was done by comparing retention times with those of external standards. Calibration was established using standard solutions of ascorbic acid ranging from 1 to 600 mg L^−1^, and peak areas were used to construct the analytical curve under the same chromatographic conditions. The results were adjusted for maltodextrin concentration and expressed on a dry weight basis.

### Preparation of extracts for antioxidant and total phenolic analyses

The extracts used for the antioxidant activity (DPPH and ABTS) and determination of total phenolic compounds (TPC) were prepared from the spray‐dried green acerola powders. For each condition, 1.0 g of sample was diluted in 100 mL ethyl alcohol (pure analytical) under constant stirring until complete homogenization. The ethanolic solution was then used as the analytical extract.^27^ The results were corrected for the dilution factor to express the values relative to the original powder mass.

### Antioxidant activity by DPPH

The antioxidant activity based on the DPPH free radical scavenging assay was determined according to the modified procedure of Brand‐Williams *et al*.^28^ For the analysis, 0.1 mL of each extract, prepared at four different dilution ratios (1:2, 1:3, 1:5, and 1:10), was added to 3.9 mL of a 60 μmol L^−1^ DPPH methanolic solution. The reaction mixtures were incubated in the dark for 2 h, a period previously established in preliminary tests to ensure stabilization of the reaction. Absorbance readings were taken at 515 nm using a UV–visible spectrophotometer (UV1600 Pro, Shimadzu Corporation, Kyoto, Japan). A standard curve (0–60 μmol L^−1^ DPPH) was generated using methanol as the blank. Antioxidant activity was expressed as EC_50_, defined as the concentration of extract necessary to decrease the initial DPPH concentration by 50%. All values were adjusted for differences in maltodextrin concentration and reported on a dry matter basis.

### Antioxidant activity by ABTS

The ABTS assay, adapted from the method described by Mareček *et al*.,^29^ was used to evaluate the antioxidant activity by comparing the ability of the sample to neutralize the ABTS^•+^ radical relative to the standard antioxidant Trolox. An ABTS stock solution (7 mmol) was prepared by reacting ABTS with potassium persulfate (K_2_S_2_O_8_) and allowing it to stand in the dark for 16 h to generate the radical cation. Prior to use, the solution was diluted with ethanol to obtain an absorbance of 0.70 ± 0.05 at 734 nm. For the assay, 30 μL of the sample (prepared at four different dilutions) was added to 3 mL of the ABTS^•+^ working solution. Absorbance was measured at 734 nm after 6 min of reaction under continuous agitation. A standard curve ranging from 100 to 2000 μmol Trolox was constructed, and antioxidant capacity was expressed as micromoles of Trolox equivalents per gram of sample (μmol TE g^−1^). The data were corrected for differences related to maltodextrin concentration and presented on a dry weight basis.

### Determination of TPC

The quantification of TPC was conducted using the Folin–Ciocâlteu colorimetric method, following the protocol described by Waterhouse,^30^ with adjustments. An aliquot of 0.5 mL of the powdered extract, previously diluted in absolute ethanol, was combined with 2.5 mL of 10% Folin–Ciocâlteu reagent and 2.0 mL of a 4% sodium carbonate solution. The reaction mixture was homogenized and kept in the dark at ambient temperature for 2 h. Absorbance readings were taken at 750 nm using a UV–visible spectrophotometer. A calibration curve was prepared using gallic acid standard solutions, and results were expressed as milligrams of gallic acid equivalents (GAE) per gram of green acerola powder. Data were corrected for the dry basis and evaluated as a function of maltodextrin concentration.

### Hygroscopicity

Hygroscopicity analysis was performed following the procedure described by Tonon *et al*.,^31^ with modifications. Approximately 1 g of the green acerola powder was placed in a tightly sealed container containing a saturated sodium chloride (NaCl) solution, which maintained a relative humidity of 75%. The system was maintained at 25 °C for 7 days, after which the samples were weighed to determine the constant mass corresponding to hygroscopic equilibrium. The hygroscopicity was then calculated as described in Eqn (1):

(1) $$\text{Hygroscopicity}\;\left(\%\right)=\left[\frac{\frac{∆m}{\left(M+M_{i}\right)}}{1+\frac{∆m}{M}}\right]\times 100$$

where Δ*m* (g) represents the mass gained by the powder after reaching equilibrium, *M* (g) corresponds to its initial mass, and *M*_*i*_ refers to the initial moisture content (free water) present in the sample prior to exposure to the humid environment.

### Morphology

The morphology of the powder surfaces was observed using a scanning electron microscope (LEO EVO 40 XVP, Zeiss, Oberkochen, Germany). The samples were fixed on aluminum stubs with double‐sided carbon adhesive and then coated with a thin layer of gold (~20 nm thickness) deposited by sputtering under a vacuum of ~10–5 torr for surface analysis of the powders.

### Statistical analysis

The outcomes derived from the CCRD were modeled using a second‐order polynomial equation that relates the response variable *Y* (including water activity, process yield, color attributes, ascorbic acid content, antioxidant capacity via DPPH and ABTS assays, TPC, and hygroscopicity) to two independent variables: drying temperature (*X*_1_) and maltodextrin concentration (*X*_2_), as presented in Eqn (2):

(2) $$Y=\beta_{0}+\beta_{1}X_{1}+\beta_{2}X_{2}+\beta_{11}X_{1}^{2}+\beta_{22}X_{2}^{2}+\beta_{12}X_{1}X_{2}$$

The regression analysis, analysis of variance (ANOVA), lack‐of‐fit tests, and generation of response surface plots were performed using the Statistica software package, version 14.1.0.8 (StatSoft Inc., Tulsa, OK, USA). Model adequacy was evaluated through the coefficient of determination (*R* ^2^) and ANOVA at a 95% confidence level. The primary section of the ANOVA table was used to perform *F*‐tests comparing the model variance to the residual variance. A model was considered adequate when the calculated *F*‐value exceeded the tabulated *F*‐value, indicating good fit to the experimental data. In cases where no statistically significant effects (*P* > 0.05) were observed, the model was not applied.

The regression equations were expressed in coded form. Optimization of the response variables was conducted simultaneously using the desirability function, as described by Santos *et al*.^32^ A validation experiment was subsequently carried out at the optimal point suggested by the software, and the tests were performed in triplicate. Comparison between the predicted and experimental values under optimal conditions was performed using Tukey's test at a 5% significance level (*P* < 0.05).

The individual desirability (*d_i_*) for each response, within the range of 0 ≤ *d_i_* ≤ 1, was calculated using Eqns (3) and (4) for the responses that were to be minimized and maximized, respectively,^33^ considering that the desirability function took into account only the parameters that were statistically significant.

(3) $$d_{i}=1,\;\text{if}\;y_{i}\leq T_{i}\left|\left(\frac{U_{i}-y_{i}}{U_{i}-T_{i}}\right),\;\text{if}\;T_{i}<y_{i}<U_{i}\right|0,\;\text{if}\;y_{i}>T_{i}$$

(4) $$d_{i}=0,\;\text{if}\;y_{i}\leq L_{i}\left|\left(\frac{y_{i}-L_{i}}{T_{i}-L_{i}}\right),\;\text{if}\;L_{i}<y_{i}<T_{i}\right|1,\;\text{if}\;y_{i}>T_{i}$$

In the optimization process, *y_i_* represents the observed response value, while *T_i_* corresponds to the target value established for that response. For variables intended to be minimized, *U_i_* denotes the upper limit, whereas, for those to be maximized, *L_i_* indicates the lower threshold. The overall desirability index (*D*) was computed by combining the individual desirability functions (*d_i_*) for each response, as illustrated in Eqn (5):

(5) $$D=\sqrt[{N}]{\left(\prod_{i=1}^{N}d_{i}\right)}$$

where *N* denotes the total number of response variables considered in the multi‐response optimization process.

## RESULTS AND DISCUSSION

Table 2 presents the results of water activity, yield, color parameters, ascorbic acid, antioxidant activity by DPPH and ABTS, TPC, and hygroscopicity of the green acerola powder under different conditions of temperature and maltodextrin concentration. Table 3 presents the *P*‐values and regression coefficients (RC) for the linear, quadratic, and interaction terms of each response variable for the green acerola powder.

**Table 2:** Water activity, yield, color parameters (L* and *h*°), ascorbic acid, antioxidant activity by DPPH and ABTS, total phenolic compounds (TPC) and hygroscopicity from the drying trials according to the experimental design of the central composite rotatable design (CCRD)

Test	*X*_1_	*X*_2_	Water activity	Yield (%)	Color parameter		Ascorbic acid (g 100 g^−1^)	DPPH (g g^−1^ of DPPH)	ABTS (μmol TE g^−1^)	TPC (mg GAE g^−1^)	Hygroscopicity (%)
					L*	*h*°					
1	120	7.0	0.283	60.93	80.00	87.54	24.30	5.67	4685.76	117.59	21.01
2	170	7.0	0.180	53.84	76.10	83.06	23.03	7.86	2934.27	161.14	22.03
3	120	26.0	0.216	53.56	80.28	85.86	24.16	13.18	2954.05	107.16	20.01
4	170	26.0	0.194	61.15	77.17	82.60	24.87	23.31	2904.05	231.80	21.72
5	110	16.5	0.227	54.56	80.06	85.97	24.09	16.34	1139.60	236.94	20.47
6	180	16.5	0.214	40.43	78.95	83.23	25.86	23.77	1399.15	238.91	21.79
7	145	3.0	0.255	39.45	78.08	86.20	21.38	0.94	3789.64	258.71	21.78
8	145	30.0	0.237	39.48	78.89	84.40	20.37	2.49	3447.52	273.02	21.10
9	145	16.5	0.190	67.97	74.43	80.89	25.70	3.48	3700.84	232.10	21.63
10	145	16.5	0.181	68.24	75.77	82.08	25.36	3.78	3713.96	234.61	21.94
11	145	16.5	0.194	68.11	73.37	79.81	24.90	3.08	3556.93	253.42	22.00

Variables: *X*_1_, temperature (°C); *X*_2_, maltodextrin concentration (%).

**Table 3:** Estimation of the *P*‐value and regression coefficient (RC) for the linear (L), quadratic (Q), and interaction terms in each response for green acerola powder

Variable	*R* ^2^ (%)	Mean		*X*_1_: Temperature (L)		Temperature (Q)		*X*_2_: Maltodextrin (L)		Maltodextrin (Q)		*X*_1_ × *X*_2_ interaction	
		RC	*P*‐level	RC	*P*‐level	RC	*P*‐level	RC	*P*‐level	RC	*P*‐level	RC	*P*‐level
Water activity	81.03	0.19	0.00	−0.02	0.05	0.01	0.21	−0.01	0.23	0.03*	0.03*	0.02	0.10
Yield	65.73	68.06	0.00	−2.43	0.50	−6.88	0.15	−0.00	1.00	−10.75*	0.04*	3.67	0.47
*L* *	86.97	74.52	0.00	−1.08	0.05	2.37*	0.01*	0.31	0.50	1.80*	0.02*	0.20	0.76
*h*°	92.35	80.93	0.00	−1.46*	0.01*	1.82*	0.01*	−0.58	0.14	2.12*	0.00*	0.30	0.55
Ascorbic acid	82.69	25.32	0.00	0.24	0.54	0.12	0.79	0.03	0.93	−1.92*	0.01*	0.49	0.38
DPPH	91.63	3.44	0.14	2.87	0.07	8.85*	0.00*	3.13*	0.05*	−0.50	0.74	1.98	0.30
ABTS	76.11	3653.84	0.00	−181.58	0.51	−970.71*	0.03*	−279.66	0.32	214.15	0.51	425.37	0.29
TPC	30.69	240.39	0.00	21.58	0.41	−26.45	0.40	10.02	0.69	−11.12	0.71	20.27	0.57
Hygroscopicity	95.64	21.86	0.00	0.58*	0.00*	−0.39*	0.01*	−0.28*	0.01*	−0.23*	0.04*	0.17	0.14

* Statistically significant coefficients (*P* < 0.05).

### Water activity (*a*_w_)

**Figure 2:**
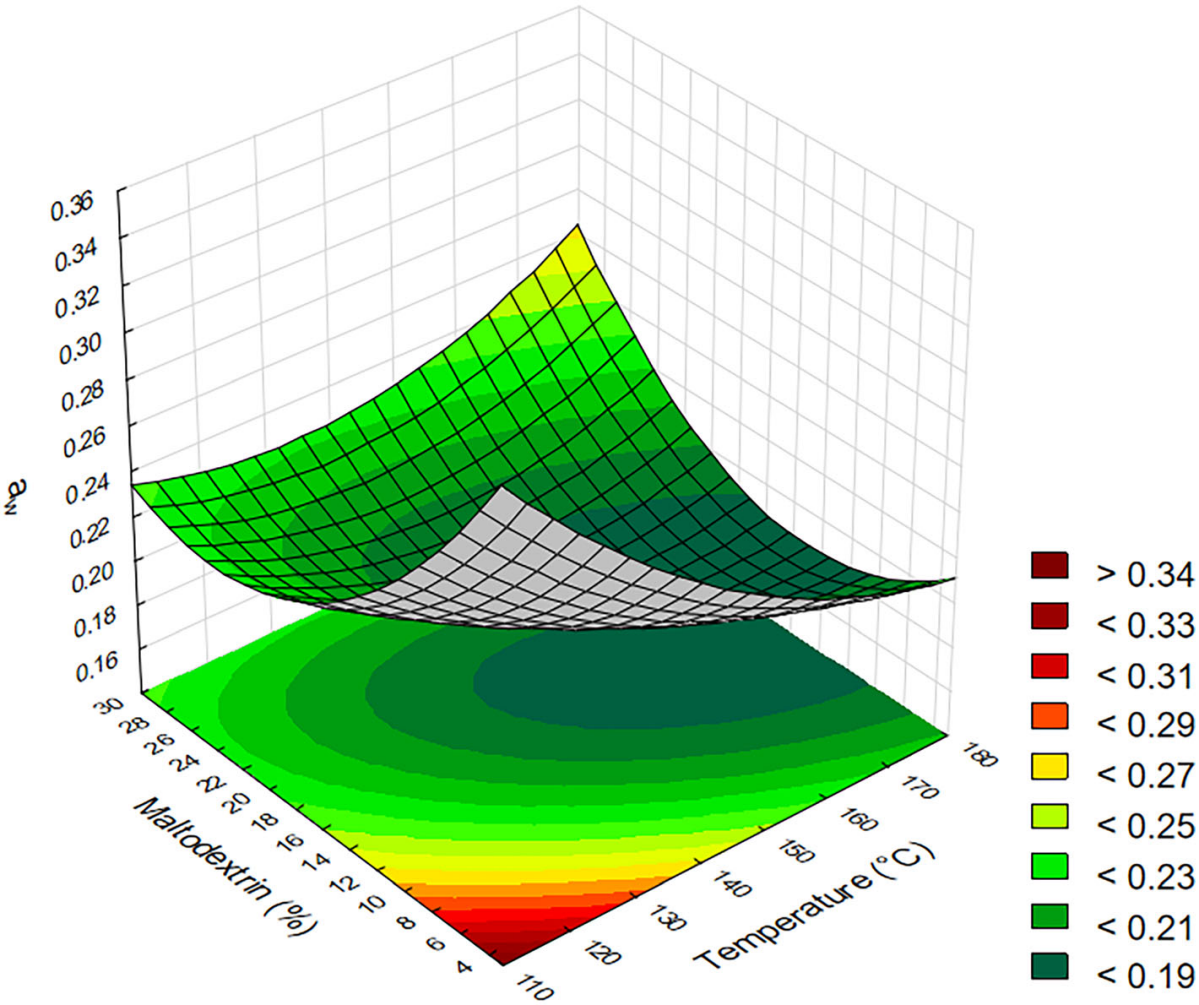
Response surface for water activity (*a*_w_) as a function of drying temperature and maltodextrin concentration.

Regarding *a*_w_, the statistically significant effect was the quadratic term of maltodextrin concentration, whose regression coefficient was positive. This indicates a convex curvature in the response surface, where intermediate maltodextrin concentrations resulted in lower *a*_w_ values. At both low and high concentrations, *a*_w_ tended to increase, as illustrated in Fig. 2. It was also observed that the effect of inlet temperature was nearly significant (*P*‐value = 0.05), suggesting that higher temperatures tend to result in lower water activity. According to Fig. 2, temperatures above 135 °C led to reduced *a*_w_, particularly when maltodextrin concentrations ranged between 12% and 22% (w/w, based on total solids content). A coefficient of determination of 81.03% was obtained, and the model provided the regression coefficient (Table 3).

For *a*_w_, the experimental values ranged from 0.180 to 0.283 (Table 2), which are lower than those reported by Ribeiro *et al*.^12^ for acerola powder, who found values between 0.35 and 0.70. The *a*_w_ is a critical parameter in drying, as high values do not ensure safety of the product.^34^ Products with *a*_w_ of 0.350 or lower are considered stable due to the limited availability of water for microbial growth and biochemical reactions. The lower the *a*_w_, the lower the water availability and its influence on the progression of reactions occurring in the product.^35^ Arepally and Goswami^36^ highlighted in their study that the carrier content significantly affects the water activity of spray‐dried powders. An increase in maltodextrin content generally led to a decrease in the *a*_w_ of the product. However, this trend was not maintained at higher inlet temperatures (180 °C) combined with elevated maltodextrin concentrations (30%). Under these conditions, the rapid formation of a rigid outer crust on the droplet surface likely limited moisture diffusion from the inner core, resulting in slightly higher water activity. In contrast, at lower maltodextrin levels, the thinner film structure promoted more efficient moisture evaporation, leading to lower *a*_w_ values at 180 °C.^18^

### Yield

ANOVA revealed that the quadratic term of maltodextrin concentration was statistically significant (*P* < 0.05), exhibiting a negative effect, revealing a convex response in which intermediate maltodextrin levels maximize yield. In contrast, temperature did not present a significant influence on the response variable, indicating that the drying process could potentially be carried out across a broader temperature interval without compromising the outcome. The model yielded a coefficient of determination (*R* ^2^) of 65.73%, which reflects a moderate fit and indicates that the regression equation provides a reasonable approximation of the actual system behavior (Table 3).

**Figure 3:**
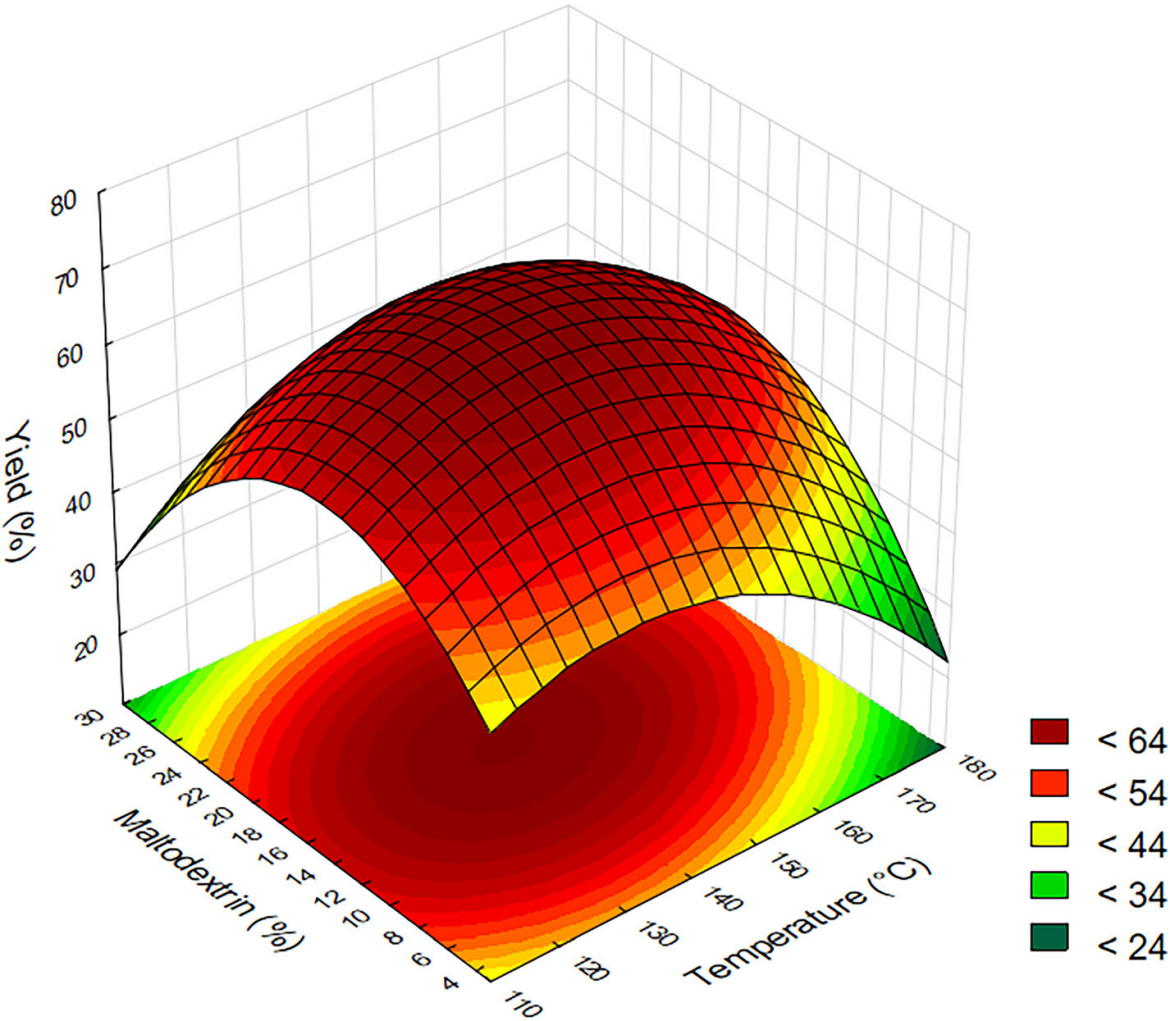
Response surface for yield as a function of drying temperature and maltodextrin concentration.

Analyzing the response surface in Fig. 3, it is evident that both very low and very high maltodextrin concentrations, approaching the axial points, reduce yield, indicating that intermediate maltodextrin levels are more favorable. At high maltodextrin concentrations, the increased viscosity of the feed solution likely impairs atomization efficiency, generating larger droplets and reducing the surface area available for moisture evaporation. This leads to incomplete drying and greater wall deposition within the spray dryer, consequently decreasing process yield.^18^ However, since maltodextrin increases the glass transition temperature of the product (*T*_g_), no significant difference was observed with temperature. The yield of the spray‐dried powder tends to increase with rising inlet air temperature, up to a certain optimal point. This behavior is closely related to the difference between the particle temperature and *T*_g_. When the inlet temperature is excessively high, the surface temperature of the particles may rapidly exceed *T*_g_, leading to adhesion of semi‐dried droplets to the inner walls of the drying chamber. On the other hand, if the temperature difference is too small, incomplete drying can also cause particles to stick, negatively affecting yield. It is also widely recognized that increasing the concentration of carrier agents, such as maltodextrin, helps reduce stickiness by elevating *T*_g_, thereby enhancing powder recovery during spray drying.^37^

High maltodextrin concentrations are known to increase the viscosity of feed solutions, which can hinder atomization and lead to the formation of larger and more agglomerated particles. As observed in Fig. 8, particularly in micrographs (a) and (c), the particles appear visibly irregular and more clustered. Although viscosity was not measured in this study, previous works have reported that increased feed viscosity can promote agglomeration and contribute to undesirable fouling within spray dryers.^31^, ^38^ During drying, particles collide with the chamber walls, and those that remain moist or sticky upon impact tend to adhere to the surface. The material deposited on the walls reduces the drying yield and increases the risk that larger powder fragments may detach and mix with the remaining product. After prolonged exposure to high temperatures, these deposits may become excessively dry, compromising product quality and potentially promoting darkening, an effect commonly associated with thermal degradation during spray drying.^39^

High yields, reaching up to 68.24%, were observed at the central points. Aryaee *et al*.,^40^ when spray drying mixed fruit juice (red grape, blackberry, and strawberry) using 20% maltodextrin (w/w of the liquid feed), reported yields of up to 51.7%. In our study, maltodextrin concentration was expressed relative to the total solids content (dry basis), which may account for the higher recovery obtained. Bazaria and Kumar^38^ obtained yields ranging from 41.31% to 54.63% in a spray drying of beetroot juice, varying the temperature and maltodextrin concentration. Braga *et al*.^41^ reported yields of up to 62.92% in a spray drying of pineapple and mint juice, varying maltodextrin concentration. Therefore, it can be concluded that the achieved yields were comparable to those reported in the literature for other spray‐dried juices.

### Color parameters L* and *h*°

**Figure 4:**
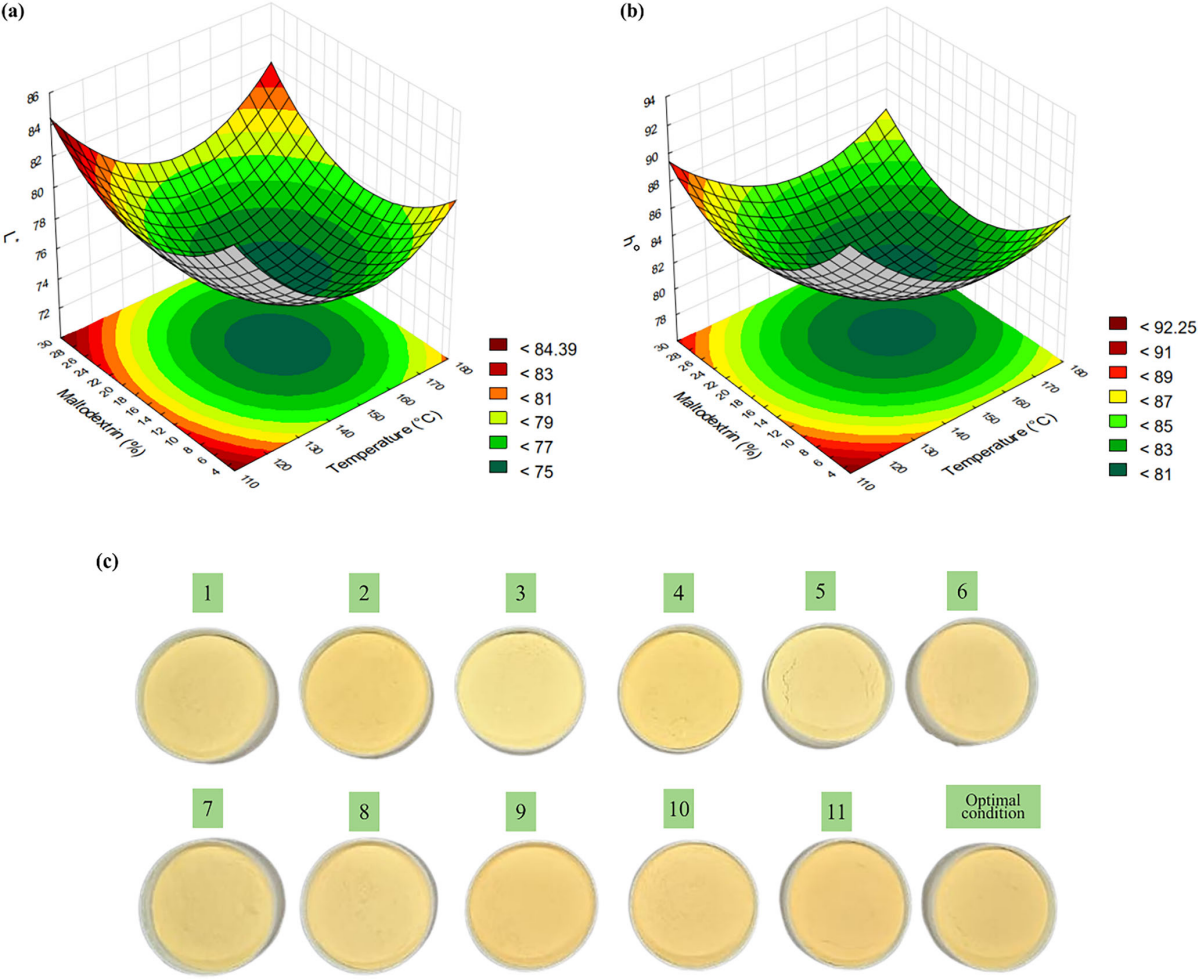
Response surface graphs for (a) lightness (L*) and (b) hue angle (*h*°) as functions of drying temperature and maltodextrin concentration, and (c) visual appearance of the spray‐dried green acerola powders produced according to the experimental design.

For the L* color parameter, the statistically significant effects were the quadratic terms for both temperature and maltodextrin concentration, with positive regression coefficients indicating a minimum point within the studied region. For the *h*° parameter, the statistically significant effects were the linear and quadratic terms for temperature and the quadratic term for maltodextrin concentration, with positive regression coefficients indicating a minimum point within the studied region (Fig. 4(a)). The coefficients of determination obtained were 86.97% for L* and 92.35% for *h*°, and the model provided the regression coefficient (Table 3).

Maltodextrin and temperature significantly affect the color parameters due to the sensitivity of pigments to the heating process and the dilution effect of maltodextrin (a white carrier material) on the color of the powders.^42^ The color differences among the experimental trials can be observed in Fig. 4(c). Caliskan and Dirim^43^ concluded that L* increased with higher maltodextrin concentrations during the spray‐drying process of sumac extracts. The decrease in L* at high inlet temperatures might be due to non‐enzymatic browning reactions, mainly Maillard reactions between reducing sugars (glucose and fructose) and amino compounds naturally present in the acerola pulp, and caramelization of sugars under intense thermal exposure.

Regarding the hue (*h*°) parameter, significant effects of both drying temperature and maltodextrin concentration were observed, indicating that these variables influenced the color tone of the green acerola powder. Observing Fig. 4(b), one can note that low maltodextrin concentrations and drying temperatures increase the product's hue, making it more yellow. This effect occurs because low maltodextrin concentrations hinder the drying process, reducing the drying rate and increasing the exposure of the product to heat within the chamber, which can promote caramelization and color darkening. Additionally, oxidation of compounds such as vitamin C contributes to this color change, which can be confirmed by analyzing yield and ascorbic acid content.^39^

### Ascorbic acid

**Figure 5:**
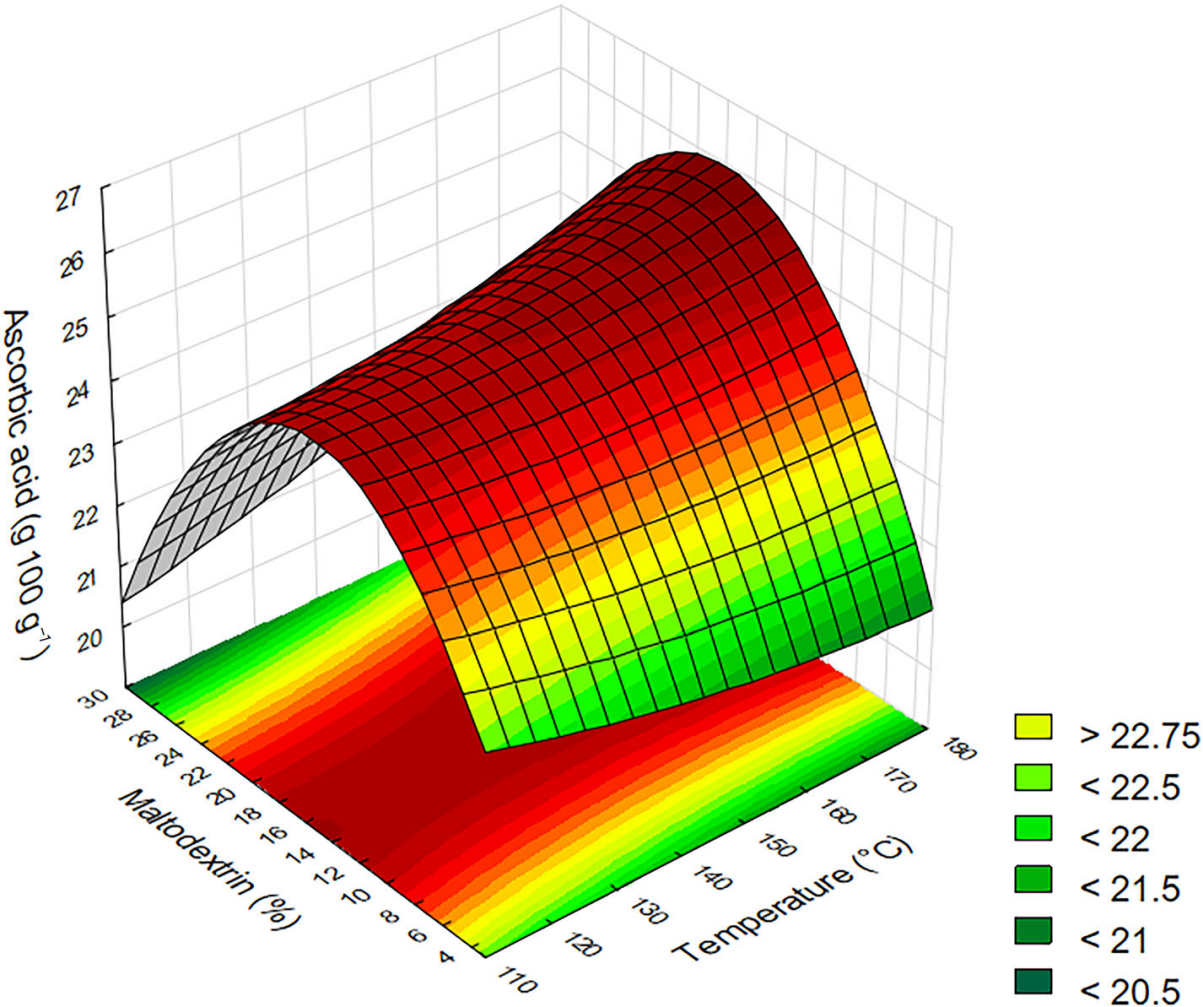
Response surface for ascorbic acid as a function of drying temperature and maltodextrin concentration.

For ascorbic acid concentration, the statistically significant effect was the quadratic term of maltodextrin concentration, with a negative effect. The relationship between temperature and maltodextrin concentration influencing ascorbic acid content presented a saddle‐shaped surface (Fig. 5), where intermediate maltodextrin concentrations favored higher retention of ascorbic acid in the powder. At very high maltodextrin levels (30%), the increased viscosity of the feed solution likely impaired atomization and moisture removal, leading to localized thermal degradation of ascorbic acid despite the greater amount of carrier material.^18^, ^44^ The model exhibited a coefficient of determination of 82.69%, and provides the regression coefficient (Table 3).

The increase in maltodextrin content, up to a certain level, led to improved ascorbic acid retention. As shown above, the best drying yield and color stability were found at intermediate maltodextrin concentrations. Therefore, it can be inferred that the presence of the carrier material contributed to protecting ascorbic acid against degradation during spray drying.^24^, ^37^, ^45^, ^46^

Previous studies have shown that the combination of inlet air temperature and maltodextrin concentration can negatively influence the retention of vitamin C in spray‐dried fruit juices, depending on the balance between heat exposure and carrier content. For instance, Lee *et al*.^47^ reported a reduction in vitamin C levels in Asian pear juice powder under such conditions. Similarly, Patil *et al*.^48^ observed that both increased drying temperature and maltodextrin content led to a decrease in vitamin C concentration in guava powder. Kim *et al*.^49^ found a comparable trend in Japanese apricot juice powder, noting a decline in vitamin C when higher amounts of maltodextrin were used alongside elevated temperatures. These effects may be associated with enhanced oxidation rates or other physicochemical changes occurring during droplet drying, such as variations in heat and mass transfer dynamics influenced by maltodextrin concentration. The decrease in ascorbic acid content is likely associated with thermal degradation and oxidative reactions, which, according to previous studies, may be enhanced by increased feed viscosity resulting from higher maltodextrin concentrations.

However, high ascorbic acid values were obtained, reaching up to 25.86 g 100 g^−1^ of powder. These values are higher than those reported by Coelho *et al*.^50^ for spray‐dried acerola juice using 15% maltodextrin, which reached 11.54 g 100 g^−1^. Braga *et al*.,^51^ when drying acerola residue in a spouted bed dryer, found up to 48.30 mg 100 g^−1^ of vitamin C. The significant increase in vitamin C concentration can be attributed to the use of concentrated juice, the incorporation of unripe acerola, known for its higher vitamin C content, and the experimental design, which optimized process parameters to improve compound retention during drying.^3^, ^4^, ^5^, ^6^

### Antioxidant activity (DPPH and ABTS)

Antioxidant analyses using DPPH and ABTS are based on measuring the ability of antioxidants to neutralize free radicals. DPPH evaluates the antioxidant's capacity to donate electrons or hydrogen atoms, while ABTS can be reduced through both electron and hydrogen donation. This makes DPPH more suitable for nonpolar samples and lipophilic compounds, whereas ABTS is more effective for both polar and lipophilic samples. However, both assays are commonly used together for a more comprehensive evaluation.^29^ In this study, a lower DPPH value and a higher ABTS value indicate greater antioxidant activity.

**Figure 6:**
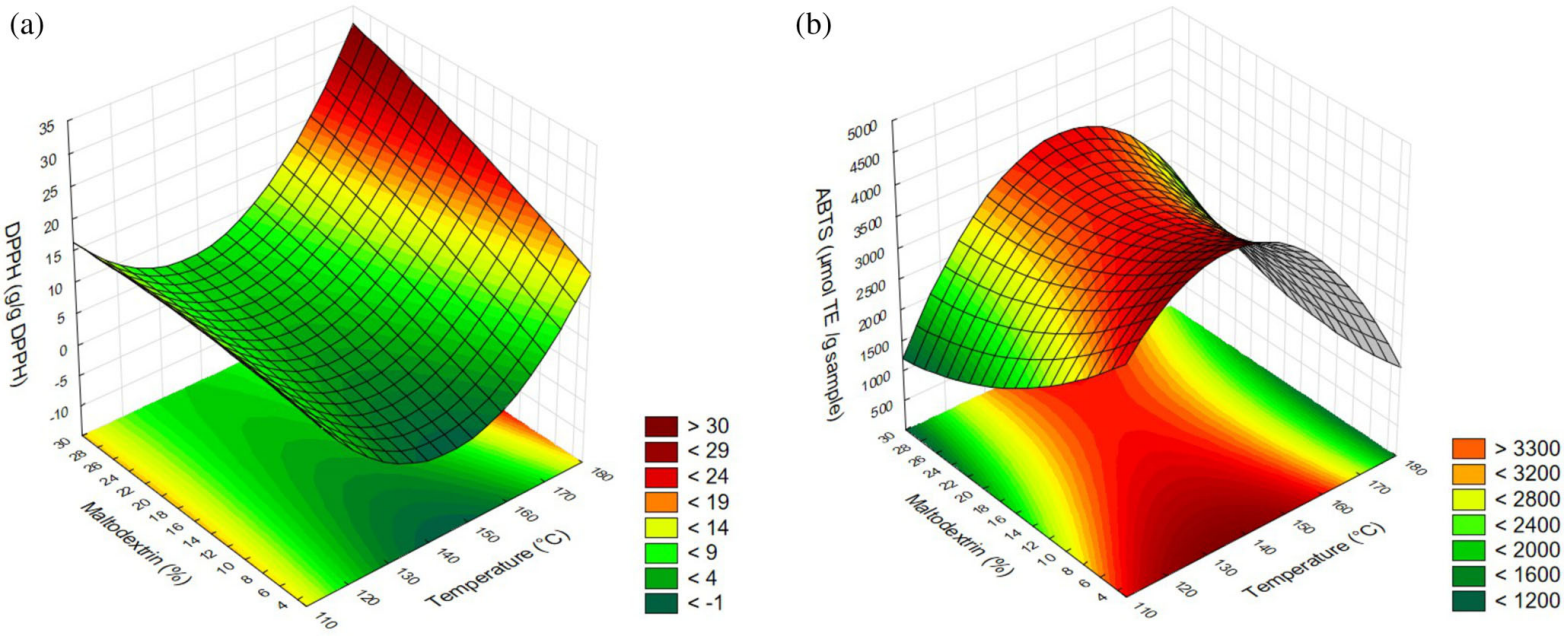
Response surface for DPPH as a function of drying temperature and maltodextrin concentration (a), and response surface for ABTS as a function of drying temperature and maltodextrin concentration (b).

Regarding antioxidant activity assessed by the DPPH method, statistically significant effects were observed for the quadratic term of temperature and the linear term of maltodextrin concentration, both presenting positive regression coefficients. This suggests that higher temperatures and maltodextrin concentrations lead to increased DPPH values. However, it is important to note that the interpretation of DPPH results is inverse, as lower sample amounts required to reduce the DPPH reagent by 50% indicate higher antioxidant capacity. Therefore, the response surface (Fig. 6(a)) indicates that antioxidant activity (DPPH) reached its lowest values at intermediate drying temperatures, consistent with the convex curvature observed for temperature, while the effect of maltodextrin concentration was nearly linear throughout the evaluated range. The quadratic temperature effect observed reflects an ideal equilibrium point. Intermediate temperatures are effective by promoting rapid water evaporation and case hardening (protective crust formation), minimizing prolonged heat exposure that would occur at low temperatures, as well as immediate thermal degradation caused by high temperatures.^52^ The determination coefficient was 91.63%, and the model provided the regression coefficient (Table 3). Regarding antioxidant activity measured by the ABTS assay, the only statistically significant effect was the quadratic term of temperature, which presented a negative regression coefficient. Therefore, intermediate to lower temperature levels were more effective in producing a powder with enhanced antioxidant activity, as observed in the response surface (Fig. 6(b)). This result, similar to that of DPPH, reinforces that the optimal temperature is crucial for protecting the bioactive compounds, as it ensures maximum matrix stability. The nearly linear effect of maltodextrin in both assays demonstrates that its primary function was structural: increasing feed viscosity and elevating the glass transition temperature of the matrix, thereby ensuring efficient encapsulation and stabilization against thermal degradation and oxidation.^52^ The coefficient of determination was 76.11%, and the model provided the corresponding regression coefficient (Table 3).

The antioxidant activity is directly related to the bioactive compounds present in the material. As observed for ascorbic acid and TPC, intermediate values of temperature and maltodextrin effectively preserve these compounds and consequently the antioxidant activity. This is attributed to the stabilizing effect of maltodextrin as a protective agent, as well as the preservation of the structure of the compounds.^37^, ^53^ Similarly, proper and efficient encapsulation preserves bioactive compounds and their associated antioxidant activity, as observed in the yield, ascorbic acid, and TPC analyses. At the experimental extremes, vitamin C oxidation likely occurred, accompanied by more irregular and clustered surface structures, as qualitatively observed in the micrographs.^39^

### TPC

The ANOVA results indicated that none of the independent variables had a statistically significant effect (*P* > 0.05) on the response variable under investigation, and the model exhibited a low coefficient of determination (*R* ^2^ = 30.69%). Given these findings, model fitting was not considered appropriate. Based on the analysis of the experimental data, it can be inferred that variations in drying temperature and maltodextrin concentration did not significantly influence the TPC of the resulting powder. However, for practical aspects, lower temperatures (below 120 °C) did not help TPC encapsulation, as tests conducted under these conditions (treatment) showed a lower TPC value. Lower drying temperatures result in powders with higher moisture content, which promotes adhesion inside the drying chamber and consequently decreases process yield.^54^ The reverse effect of high temperature on TPC content can also be observed in assay 2, conducted at 170 °C. Gupta and Banerjee^55^ suggested that heat can structurally alter polyphenolic compounds and bind polyphenols to other compounds, leading to a reduction in their total content. Temperature extremes combined with low wall material concentration result in decreased TPC preservation.^53^

The relatively low *R* ^2^ obtained for TPC suggests that other process or matrix‐related factors, not included in the present experimental design, may have contributed to the variability of phenolic content. This behavior may be attributed to the intrinsic variability of phenolic compounds in acerola juice, their high susceptibility to oxidative degradation during atomization, and potential influences of operational parameters such as feed flow rate, inlet air humidity, or droplet size. These factors, although not assessed in this study, could have modulated the exposure of the phenolics to oxygen and heat, thereby affecting their recovery. Future studies incorporating these variables may enable a more comprehensive modeling of TPC behavior during the spray drying of green acerola juice.

### Hygroscopicity

For hygroscopicity, the effects of temperature and maltodextrin concentration for both linear and quadratic terms were statistically significant. The regression coefficients for the quadratic terms were negative, indicating a maximum point within the study region. A coefficient of determination of 95.64% was obtained, and the model provided the regression coefficient (Table 3).

**Figure 7:**
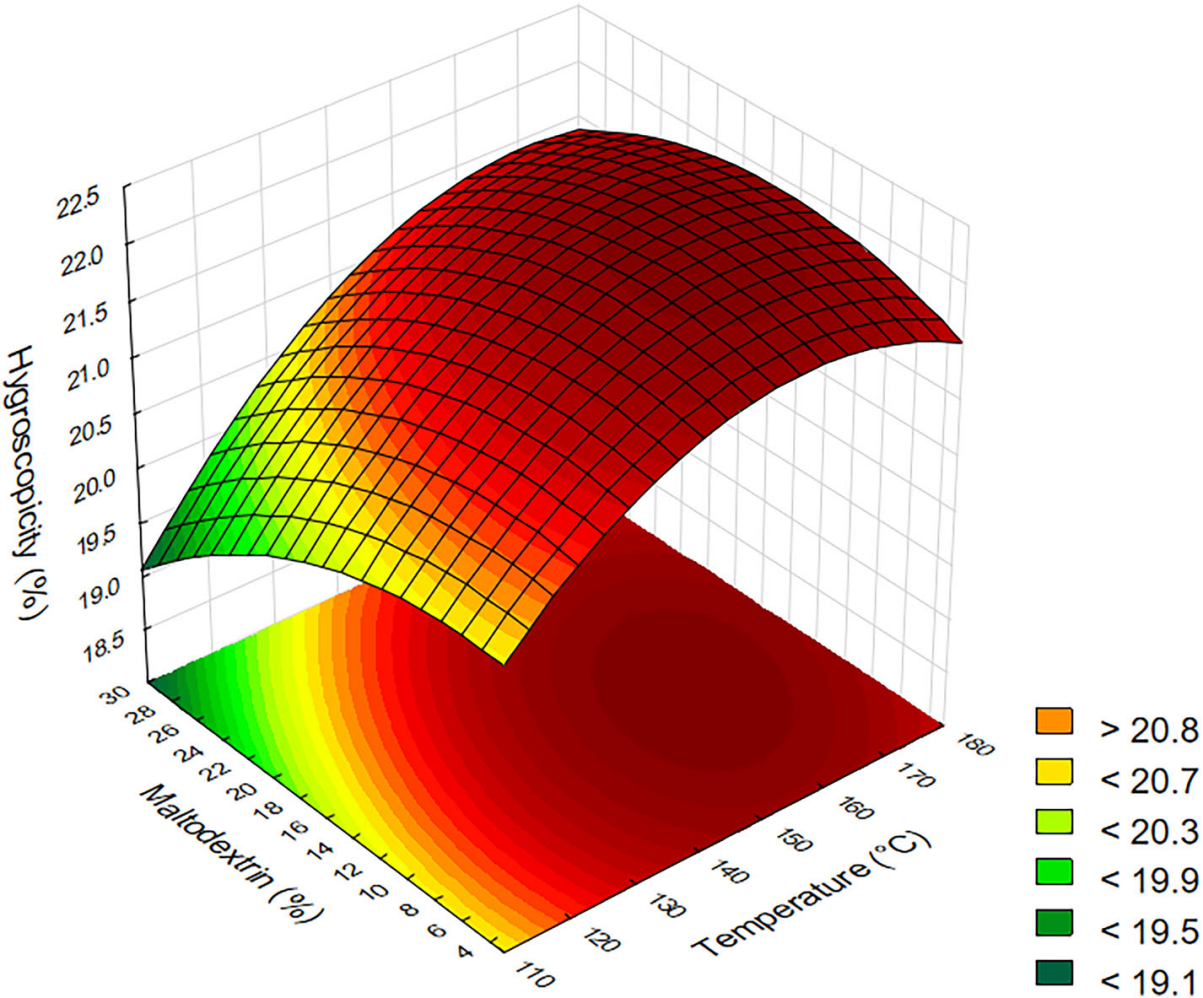
Response surface for hygroscopicity as a function of drying temperature and maltodextrin concentration.

Upon observing the response surface in Fig. 7, it is evident that lower hygroscopicity, which is desirable for this product, can be achieved with higher maltodextrin concentrations and lower drying temperatures. At higher maltodextrin concentrations (≥30%), the formation of a denser amorphous matrix reduces water adsorption by limiting the exposure of hygroscopic solutes to ambient moisture. This effect, observed in the spray‐dried powders, results in a decreased water adsorption capacity, which is beneficial for preserving product quality during storage.^56^

A result similar was found by Lima *et al*.,^11^ who also observed a relationship between the decrease in hygroscopicity and the increase in maltodextrin concentration. Carrier agents such as maltodextrin are recognized in spray drying for increasing *T*_g_ and yield percentage while reducing stickiness and hygroscopicity of the powder.^57^

It can be stated that powders characterized by low hygroscopicity and *a*_w_ are considered more stable and of higher quality.^58^ However, Bazaria and Kumar^59^ observed that moisture content affects the hygroscopicity of a material. The lower the moisture content, the higher the hygroscopicity, since the gradient of chemical potential of water between the product and the surrounding environment is greater, providing a stronger driving force for mass transfer. This can be observed in the present study by comparing Figs 2 and 7.

### Morphology

**Figure 8:**
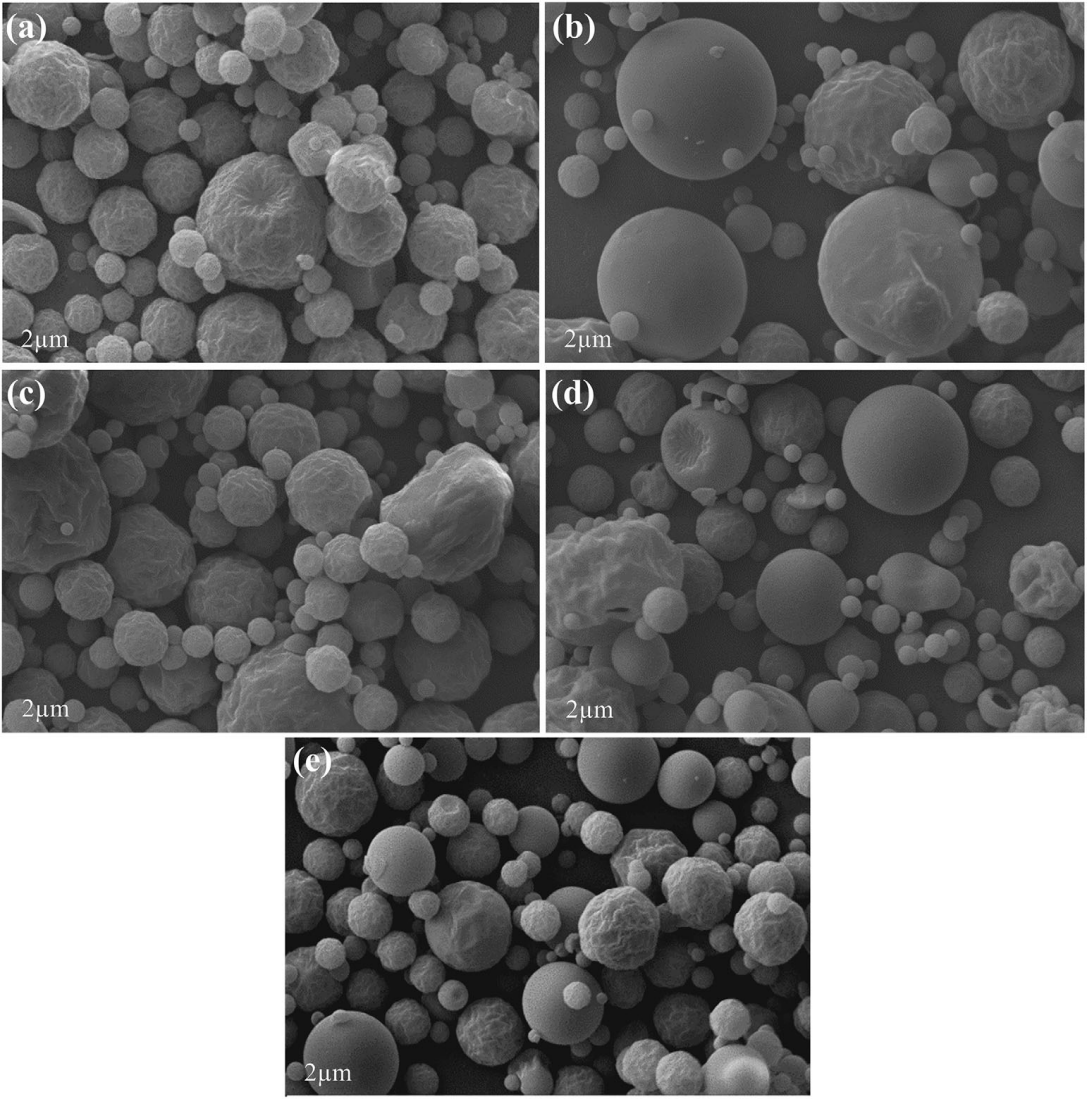
Scanning electron microscopy of trials: (a) 7% maltodextrin at 120 °C; (b) 7% maltodextrin at 170 °C; (c) 26% maltodextrin at 120 °C; (d) 26% maltodextrin at 170 °C; and (e) 16.5% maltodextrin at 145 °C).

The micrographs in Fig. 8 highlight the effect of inlet temperature and maltodextrin concentration on the morphology of spray‐dried green acerola powders. Figure 8(a),(b) correspond to trials with 7% maltodextrin, dried at 120 and 170 °C, respectively. At 120 °C (Fig. 8(a)), the particles appeared more wrinkled and irregular, whereas at 170 °C (Fig. 8(b)) larger and relatively smoother particles were formed, although with partially collapsed surfaces. A similar trend was observed in Fig. 8(c),(d) (26% maltodextrin at 120 and 170 °C, respectively).

In Fig. 8(e) (145 °C and 16.5% maltodextrin, central points), the particles exhibited more uniform sizes and smoother surfaces, indicating a better balance between inlet temperature and carrier concentration. Overall, Fig. 8(a),(c),(e) display similar morphology, characterized by numerous rough particles, whereas Fig. 8(b),(d) show a lower particle density and continued microstructural collapse.

No clear agglomeration phenomena were observed, suggesting that the process conditions effectively minimized stickiness during atomization. The morphological differences observed are mainly related to the evaporation rate and crust formation dynamics: higher inlet temperatures accelerate drying and promote rapid surface solidification, which may lead to localized deformations.^41^ In addition, a possible increase in feed viscosity may have contributed to material buildup on the chamber walls and a reduction in process yield.^31^, ^38^, ^39^ The addition of maltodextrin contributes to the formation of more stable and less adhesive particles, corroborating the yield and bioactive retention results, with the intermediate condition (145 °C and 16.5%) being the most favorable for powder stability.^41^

### Optimization

**Figure 9:**
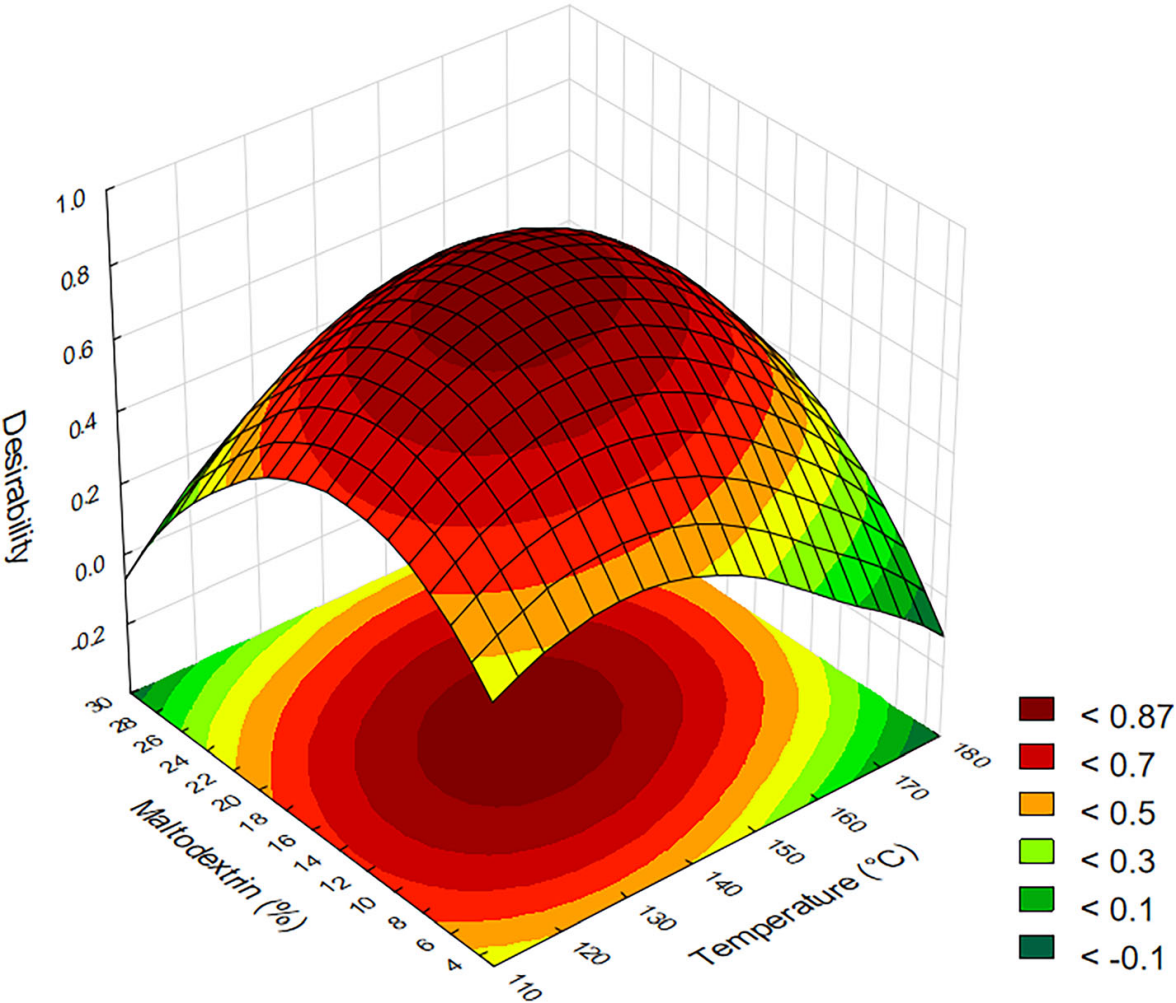
Optimal response surfaces of green acerola juice as a function of temperature and maltodextrin based on the responses obtained.

The response surface generated from the optimization process and desirability analysis, where values close to 1.0 indicate more favorable outcomes, suggests that intermediate conditions of drying temperature and maltodextrin concentration provide the most suitable parameters for processing green acerola juice. The optimal point was identified at approximately 145 °C and 16.5% maltodextrin, as shown in Fig. 9. The predicted and experimentally observed values under this optimal condition are presented in Table 4.

**Table 4:** Predicted and experimental values under the optimal spray‐drying conditions for green acerola powder

Response	Predicted value	Experimental value (mean ± SD)
Water activity	0.180a	0.181 ± 0,003a
Yield (%)	68.240a	76.000 ± 2.000b
L*	80.280a	78.190 ± 3.154a
*h*°	87.540a	83.250 ± 3.309a
Ascorbic acid (g 100 g^−1^)	25.860a	25.240 ± 0.403a
DPPH (g g^−1^ of DPPH)	0.940a	1.190 ± 0.287a
ABTS (μmol TE g^−1^)	4685.800a	4201.180 ± 463.186a
Hygroscopicity (%)	20.010a	20.020 ± 0.411a

SD, standard deviation. Means followed by the same letter do not differ significantly according to Tukey's test (*P* < 0.05).

The experimental values were very close to the predicted ones for water activity, color parameters (L* and *h*°), ascorbic acid content, DPPH, ABTS, and hygroscopicity, showing no statistically significant differences (*P* > 0.05). Only the yield differed significantly from the predicted value, however, it was higher than expected, demonstrating the adequacy of the model and the effectiveness of the selected drying conditions. Therefore, it can be concluded that the model is highly suitable for evaluating the behavior of the spray‐drying process of concentrated green acerola juice. Spray drying is an efficient method for encapsulating and preserving bioactive compounds. The combination of high temperatures with the inherently short drying duration characteristic of spray drying makes this technology effective for preserving sensitive materials.^10^

However, extreme values of temperature and maltodextrin concentration can negatively affect the drying process of green acerola. As observed in Fig. 9 and throughout the discussion, intermediate values are more effective concerning the analyzed responses, such as yield, bioactive compounds, and the physical characteristics of green acerola powder. This highlights the importance of process optimization and the correlation between the analyzed variables (temperature and maltodextrin) and the responses of interest. The use of maltodextrin as a wall material, at appropriate concentrations, provides a stabilizing and protective effect, helping to maintain the structural integrity of the compounds.^53^ Similarly, maintaining an adequate drying temperature improves process performance and contributes to the preservation of bioactive compound structures.^53^, ^54^

## CONCLUSIONS

Response surface methodology was demonstrated to be a valuable tool for assessing the influence of drying temperature and maltodextrin concentration (expressed on a dry basis) on various quality attributes of spray‐dried green acerola powder, including process yield, ascorbic acid content, antioxidant capacity (DPPH and ABTS), water activity, hygroscopicity, and the color parameters L* and hue angle (*h*°). The adjusted regression models showed satisfactory predictive capability, with coefficients of determination (*R* ^2^) indicating good agreement between experimental and estimated values, reaching up to 95.64% for hygroscopicity. The optimal conditions for achieving the desired responses were a drying temperature of 145 °C and a maltodextrin concentration of 16.5% (dry basis). This demonstrates that intermediate levels of temperature and maltodextrin percentage are the most suitable for spray drying green acerola powder, significantly impacting high yield values, the preservation of bioactive compounds such as ascorbic acid, ensuring antioxidant activity, and the physicochemical stabilization of the green acerola powder.

## FUNDING INFORMATION

This work was financially supported by the Coordenação de Aperfeiçoamento de Pessoal de Nível Superior – CAPES, for the doctoral scholarships (Grant No. 88887.686613/2022–00), Fundação de Amparo à Pesquisa do Estado de Minas Gerais – FAPEMIG (APQ‐01076‐24), and Conselho Nacional de Desenvolvimento Científico e Tecnológico – CNPq (420009/2021‐3).

## CONFLICT OF INTEREST

The authors declare no competing interests.

## AUTHOR CONTRIBUTIONS

FS Costa: conceptualization, investigation, methodology, data curation, and writing. JCC Santos: investigation, methodology, data curation, formal analysis, validation, visualization, writing, review, and editing. MDC Silva: investigation and methodology. MS Cruz: investigation, writing, and review. AAL Santos: investigation, writing, and review. CR Oliveira: investigation, methodology, and data curation. LF Oliveira: conceptualization, investigation, data curation, formal analysis, visualization, writing, review, and editing. JLG Corrêa: conceptualization, funding acquisition, supervision, and review.

## Data Availability

The data that support the findings of this study are available from the corresponding author upon reasonable request.
